# Self-worth and life meaning: the perceived narrative frames on the identity of people with disabilities

**DOI:** 10.3389/fpsyg.2026.1798493

**Published:** 2026-09-09

**Authors:** Yawen Huang, Liu Yang, Changjiang Hu, Zhonghao Zhao

**Affiliations:** 1 School of New Media, Financial and Economic News, Guangdong University of Finance, Guangdong, China; 2 School of Journalism and Communication, Sun Yat-sen University, Guangzhou, China

**Keywords:** digital platforms, disability models, disability narratives, identity, media frames

## Abstract

This study investigated the narrative frames of disability and their associations with disability identity in the context of China. Specifically, it explored the sense of Self-worth, Life Meaning, Perceived Discrimination, and Disability Denial in people with disabilities and the complex relationships between media narratives, impairment, and social constraints. Through framing analysis, this study found three tragedy narrative frames (*traditional, medical*, and *charity*) and three non-tragedy frames (*inspirational, relationship*, and *rights*) from the traditional media and social media. Additional hierarchical regression analysis of a survey data among people with disabilities revealed that participants who frequently accessed social media platforms were more likely to reject tragic narratives while accepting non-tragic ones. The findings indicate that access to non-tragic narrative resources may help people with disabilities challenge tragedy-based understandings and construct more positive identities. Further analysis, however, indicated that superficial positive valence does not itself constitute affirmative identity acceptance. For example, although the inspirational frame celebrates agency and achievement, it retains a deficit-oriented logic in which impairments must be overcome; accordingly, it reinforces greater disability denial, thereby limiting the formation of a genuine affirmative disability identity.

## Introduction

Media are charged with telling stories and making meaning (Johnstone, 2004). These stories usually draw on different disability models that define disability and position people with disabilities in particular ways. Traditional media channels, such as novels, newspaper stories, or television and films, are often structured hierarchically. They have been portraying people with disabilities as victims, medical maladies, objects of charity, and deviants (Johnstone, 2004). These negative images were reinforced by the medical notion of disability as an adaptation to a tragedy or a mechanism to cope with stigma and personal impairment (Oliver, 1990). For people with disabilities, powerful stories leave lasting impressions. If people with disabilities internalize this stereotype, they might “come to believe they are less capable than others” (Charlton, 2010; Gibson et al., 2018).

However, beginning in the 1970s, non-tragic disability narratives grounded in the social and affirmation models emerged, reframing disability in terms of social barriers, rights, and positive identity rather than personal tragedy (Oliver, 1990; Swain and French, 2000). These alternative narrative frames offered people with disabilities new resources through which to reinterpret themselves and construct more positive identities, rather than viewing themselves primarily through impairment. Yet such narrative frames remain unfamiliar to many people with disabilities, particularly in non-Western societies (Lin et al., 2024). Traditional media were also slow to reflect this shift, as entrenched production routines, a preference for emotionally compelling stories, and the limited participation of people with disabilities in content production encouraged continued reliance on conventional tragedy-oriented narratives (Lin et al., 2024).

Over the past three decades, emerging information and communication technologies (ICTs), particularly social media, have transformed how people with disabilities access information, communicate, and build communities (Gelfgren et al., 2022). Platforms such as Facebook, Instagram, Twitter, YouTube, and Snapchat enable disability communities and advocacy groups to exchange information, voice concerns, raise public awareness, and participate in public debate. Beyond facilitating communication, these platforms provide socio-political spaces in which advocates can challenge established offline narrative structures and circulate alternative disability narratives in accessible digital forms (Gelfgren et al., 2022). By connecting people across traditional narrative boundaries, these practices can expand the narrative resources available to people with disabilities and potentially reinforce more positive identities (Castells, 2000; Gelfgren et al., 2022; Rainie and Wellman, 2012).

Traditional media institutions and disability advocates on digital platforms therefore construct disability through different narrative patterns and play distinct roles in shaping disability-related agendas. Yet little is known about whether, and to what extent, narratives produced by disability advocates on digital platforms contribute to positive identity formation among people with disabilities, particularly when compared with traditional media narratives. To address this concern, this study examined representations of disability across traditional media and digital platforms, together with the lived experiences of people with disabilities, through the lens of identity, combining framing analysis with a survey of people with disabilities.

### Disability models and identity construction

The disability model is a paradigm or perspective related to a social condition of disability as well as a tool to understand disability identity and its implied stories (Seligman and Darling, 2013). In recent decades, models and understandings of disability have changed the landscape of identity development for people with disabilities (Evans et al., 2017; Gibson et al., 2018). Certain models of disability have shaped the construct of disability identity since the Middle Ages, beginning with the moral model (disability as a consequence of a violation of morality). In the nineteenth century, the evolution of disability identity moved to a medical model (disability as a disease that needs to be cured). In the 1960s and 1970s, the development of disability identity moved to a social model (disability as a social construct created by social norms) and to an affirmation model (disability as a positive identity).

The moral model is the oldest model of disability and refers to the attitude that people are morally responsible for their own disability (Pardeck, 2012). According to one of the primary forms of this model, disability should be regarded as a punishment from God for a particular sin or sins that may have been committed by the person with a disability (Retief and Letšosa, 2018). This model considers not only the individual's sins as a possible cause of their disability but also their parents' and ancestors' sins (Henderson and Bryan, 2011). For example, disability may be seen as a consequence of immoral actions by parents if it is congenital, or as a consequence of practicing witchcraft if it is acquired (Retief and Letšosa, 2018). By highlighting how some forms of discursive interpretations such as “blindness,” “lameness,” “deafness,” “mental illness,” and other forms of disability are associated with human sin, evil, or spiritual incapacity, the moral model directly or indirectly excludes people with disabilities from the discourse of mainstream society (McClure, 2007).

Beginning in the mid-1800s, the medical model of disability gradually began to replace the moral model. Although medical science made significant advances in understanding the causes of disability (Retief and Letšosa, 2018), it conceptualized disability as undesirable and stigmatized (Goffman, 1956; Zapata, 2019) and as an impediment to be prevented, remedied, and, if possible, cured (Carlson, 2010; Kasser and Lytle, 2005). According to the medical model, people with disabilities deviate from normalcy, and terms such as “invalid,” “cripple,” “spastic,” “handicapped,” and “retarded” are all derived from this model of disability (Creamer, 2009). As disability scholars state (2012), “The medical model of interpretation of disability projects a dualism which tends to categorize the able-bodied as somehow ‘better' ‘normal' or superior to people with disabilities” (Woods and Thomas, 2003; Johnstone, 2012). Disability is thus seen as a biological problem that lies within the individual; that is, to become a full human being again, the person with a disability must undergo medical treatment and physical rehabilitation (Oliver, 1990).

Another influential framework is the charity model, which depicts people with disabilities as victims of circumstance who deserve pity (Rialland, 2006). The situation of people with disabilities is tragic, and they are suffering. People without disabilities should therefore assist people with disabilities in every way possible, as “they need special services, special institutions, etc., because they are different” (Duyan, 2007; Rialland, 2006). Since people with disabilities are considered tragic victims in this model, it follows that they need care, are unable to tend to themselves or manage their own affairs, and need handouts to survive (Lim and Chia, 2017). The charity model thus has been traditionally been used by charities in the competitive business of fund-raising, in which people with disabilities are depicted alongside other “victims” of famine, poverty, child abuse, or other tragic circumstances (Rialland, 2006). Thus, the charity model is condemned by its critics as obstructive and the cause of significant discrimination. Some even go so far as to call it a means to maintain the flow of donations to favor people without disabilities (Rialland, 2006).

Inspired by the disability movement in the 1960s and 1970s, the social model of disability developed in response to the limitations of the moral, medical, and charity models of disability (Marra, 2012). Fundamental to the social model of disability is the notion that disability is a socially constructed phenomenon rather than a physical impairment (Retief and Letšosa, 2018). Disability characterized by the social model is imposed on top of physical impairments in terms of the way people with disabilities are unnecessarily isolated and excluded from full participation in society, especially how “the physical and social environment impose limitations upon certain categories of people” (Retief and Letšosa, 2018). Compared to other models of disability, the social model is a modified representation of the world that allows disability to be viewed as a relationship of oppression or a structure of domination rather than an individual limitation (Cameron, 2014). The social model provides an analysis of disability as a social relationship and allows for a discussion of disability in terms of what people with impairments cannot do and be due to physical and social barriers (Cameron, 2014).

The affirmation model is a non-tragic view of disability and impairment that embraces positive social identities (Swain and French, 2000), begins with the acceptance that people with impairments are different, but there is nothing wrong with them that needs to be fixed (McCormack and Collins, 2012). The affirmation model is rooted in the social model but focuses more on self-acceptance within heterogeneous social environments. This view has emerged in direct opposition to the prevailing personal tragedy model of disability and impairment and builds on the liberating imperative of the social model (Swain and French, 2000). The social, political, and emotional experiences of people with impairments link them together and create a recognizable minority group called “people with disabilities” (Rialland, 2006). The affirmation model has played a significant role in inspiring persons with disabilities to adopt a positive self-image (Swain and French, 2000) and to consider disability in terms of what people with impairments are required to do and be instead (Cameron, 2014).

Conceptualizing disability using a model is important to situate the historical conditions under which it has been understood and categorized (Brighton et al., 2021). The review of the above literature reveals that models of disability follow two philosophical logics: the individualized personal tragedy and the socially constructed oppressive social environment. Disability has historically been viewed through the individualized perspective of the moral, charity, and medical models, which conceptualizes disability as undesirable and stigmatized and holds people with disabilities accountable for adopting the norms of the majority (people without disabilities) as closely as possible. The problem with this individualized narrative, as with professional constructions, is that it ignores issues of social prejudice and institutional discrimination (Oliver, 1990). The socially constructed perspective of the social and affirmation models evolved from the proposition that society is responsible for accommodating the differences of people with disabilities. It is more than merely the functional limitations an individual has or the social limitations they encounter; it is a complex relationship between impairment, social limitations, and disability identity (Oliver, 1990; Oliver et al., 1988). Therefore, the experience of disability should be situated within a broader social context and social forces (Oliver, 1990).

### Disability narratives and identity in mass media

The media help us give meaning to our lives and identities and the world around us by telling stories and conveying meanings. The media's focus on portraying people with disabilities as newsworthy is when they are portrayed as pitiful or as victims, or conversely, as objects of curiosity or as superheroes. People with disabilities are constantly interpreted as struggling, disadvantaged, or sick victims, with disability portrayed as passive and challenging (Brooke, 2019); or as someone overcoming their disability in ways that the public often views as inspiring (Oliver, 1996). Disability studies thus often refer to this type of narrative in mass media as the “pity/heroism dichotomy” (Ralston and Ho, 2009).

The pity narrative imposes a highly ordered yet highly emotional expression on the lives and limitations of people with disabilities, interpreting them as struggling, disadvantaged, or sick victims and portraying disability as passive and challenging (Brooke, 2019). A pity story may begin with a prospective mother who wants a normal healthy baby while a routine scan reveals that her child would be born with disabilities; or, it may begin with an innocent person struck by disaster, who is told by doctors that he would never walk again, realizes he would be a burden on the family, and contemplates suicide (Lin and Yang, 2018). According to these narratives, disability is a curse, a physical impairment, and a tragic story associated with the attribution of shame to the individual or family (Niemann, 2005).

Extending this construction of disability as personal tragedy, the pity narrative depicts people with disabilities as needing care, being unable to look after themselves or manage their own affairs, and depending on charity (Rialland, 2006). It may also portray them as requiring medical treatment and physical rehabilitation to become fully human again, even when their impairments are incurable (Oliver, 1990; Pfeiffer, 2003). As Nabil Shaban observed, “the non-disabled are only comfortable when you see us as icons of pity”; yet, for people with disabilities to participate fully in society as equal citizens, they require respect rather than pity (Rialland, 2006).

On the contrary, the heroism narrative in mass media portrays people with disabilities as heroes who, despite experiencing tragedy, bravely struggle against the adversity they face over the course of the narrative (Yang and Lin, 2025). For example, a person is born with multiple disabilities, and yet, they are determined that their life would be as normal as possible. The person with disabilities tries a number of conventional and alternative therapies in an attempt to get well; then, they establish a charity to raise money to be able to consult an extremely expensive magician who they consider as their last hope in their struggle for normality. They say “I will walk again!” and “Even if it kills me!” (Yang, 2020), suggesting that the hero may finally overcome adversity or at least find some compromise in which they might live happily ever after—despite their afflictions. Occasionally, they lose the battle and die.

Although more positive in tone, the heroism narrative remains grounded in a tragedy-based understanding of disability. It emphasizes overcoming tragedy through courage and determination (Schantz and Gilbert, 2001; Schell and Duncan, 1999; Smith and Thomas, 2005). Expressions such as “people with disabilities overcome adversity” and “achieve goals despite their circumstances” are common in the press, with “adversity” referring to a medical diagnosis, tragedy, accident, or discriminatory circumstances (Brooke, 2019). The narrative therefore implies that, through hard work, courage, and determination, people can demonstrate physical, sensory, or intellectual abilities beyond those expected of a person with a disability and thereby live a “normal” life (Brighton et al., 2021; Hardin and Hardin, 2004; Howe, 2011; Silva and Howe, 2012).

This pity/heroism dichotomy exemplifies the selective representational logic through which mass media construct disability. By rendering particular dimensions of disability salient, notably dependency, suffering, impairment, or exceptional achievement, while relegating structural barriers and ordinary lived experience to the discursive background, media produce patterned interpretations of disability (Lin et al., 2024). These interpretive configurations constitute media frames. Such frames operate as recurrent narrative structures through which disability is problematized, causally explained, morally evaluated, and associated with particular responses (Entman, 1993; Lin et al., 2024).

### Paradigm shift in digital platforms

Since the 1970s, with the development of the disability movement, disability has become a specific site of formative power, discourse, and apparatus (Oliver, 1996). Since then, disability studies scholars have shifted their attention from individual impairment, illness, functional disability, or psychological deviance to how a “disabling society” contributes to the recognition of people with disabilities (Barnes and Mercer, 2004). As Goggin and Newell (2000) argue, “disability can thus be viewed as a constructed socio-political space determined by prevailing norms, values encountered in technological systems, and its social context” (p. 128). The new paradigm not only has implications for the way physical structures and institutional norms are created and maintained, but can also create additional opportunities for people with disabilities to feel good about themselves.

Traditional media channels have often been established for decades and are usually hierarchical. As mentioned earlier, the literature on this population usually views disability as a form of deviation from the ability and appearance norms of mainstream society (Seligman and Darling, 2013). With this in mind, one way to support the exploration and development of identity for people with disabilities is to provide opportunities for people with disabilities to feel supported and learn about positive disability experiences (Forber-Pratt et al., 2020). More recently, with the development of the disability movement and information technology, people with disabilities have begun to work to promote disability culture, both through traditional media and the Internet (Johnstone, 2004). Digital platforms in particular, such as web pages, listservs, chat lines, and information sites, are ways in which people with disabilities have institutionalized the notion of identity and culture in mainstream society (Johnstone, 2004).

Especially, studies have shown that social media is a site of recreation and reinforcement of numerous negative attitudes toward disability, shifting from the traditional narrative of disability to diversified discourses through self-representation, debate, and discussion by people with disabilities who advocate for these narratives (Brittain, 2004). For example, people who are blind from birth might understand their blindness as a neutral way of being. As such, impairment may or may not be met with a negative evaluation by its possessor; it is the lack of barrier-free facilities that causes an individual to have disabilities (Goering, 2015). Accordingly, new narratives emphasize modifying physical and social arrangements and institutional norms to meet individual needs, for example, replacing stairs with ramps, auditory-only data presentation with multimodal communication, and restrictive work requirements with accommodations for different ways of working (Yang and Lin, 2025). Such accommodations enable people with disabilities to perform activities that matter to themselves and society and to recognize their value as equal to that of people without disabilities.

While some focus on reclaiming the body's relationship to society and the environment, others openly affirm their pride in their disability. Much like the phrase “black is beautiful” has changed the way African-Americans view themselves and their image, pride in disability helps one see the beauty in disability (Fleischer and Zames, 2000). Most people do not originally desire to have disabilities. Many claim they are important despite their disability (Fleischer and Zames, 2000; Johnstone, 2004). Pride encourages people with disabilities to “claim” their disability rather than deny or mask it, and there is nothing evil or wrong with having disabilities. Consider Deborah Kent, who reports, “To me, Disability Pride is many things: it is a chance for people with disabilities to acknowledge their self-worth, something not often done by people outside the community” (Dunn and Burcaw, 2013). Constructive acceptance of one's life situation, then, can solidify the meaning of disability while promoting a positive disability identity and often leads to the discovery of a “silver lining” (Dunn and Burcaw, 2013).

In several ways, social media have increasingly changed how people with disabilities share information, express their views, participate in public debate, and raise public awareness of disability-related issues (Gelfgren et al., 2022). The representation of people with disabilities in social media has been consistently expressed or interpreted with the logic of the theoretical pattern of the social model and the affirmation model. The identity of people with disabilities is the result of such a non-tragic narrative, which has largely led to a shift from the individualized models to a social structure of disability. It is what Peters and Chimedza (2000) have expressed as “reclaiming the body” (p. 248), which appreciates when people say, “I never think of you as disabled, I just think of you as you.” (Cameron, 2014; Michalko, 2002).

### Research question

Social media have broadened the contexts in which disability identities are represented and negotiated (Johnstone, 2004). By mirroring, modeling, and acknowledging available narrative resources (Forber-Pratt et al., 2017), people with disabilities may interpret disability differently and enact their identities through everyday experiences across social media platforms (Johnstone, 2004). Although previous studies have documented diverse understandings of disability (Johnstone, 2004), few studies have examined how narratives encountered through comparative media sources relate to disability identity. Especially, more research is examined in non-Western contexts, where disability identities may be shaped by distinct sociocultural conditions in non-Western societies, such as China (Zapata, 2019). This study thus would like to explore the narrative frames characterizing disability-related representations in traditional Chinese media and on social media. It further examines how these frames are associated with different dimensions of disability identity among people with disabilities in China.

## Methodology

This study adopted a two-stage sequential design combining a disability-model-guided framing analysis with a cross-sectional questionnaire survey. In Stage 1, we analyzed disability-related content from two traditional media outlets and three digital platforms to identify narrative frames and translate them into candidate survey items. In Stage 2, we surveyed Chinese people with disabilities to assess their perceptions of these frames and examine their associations with four dimensions of disability identity, together with sociodemographic and media-use characteristics. The questionnaire also measured participants' media use, sociodemographic characteristics, and identity-related outcomes, including Self-worth, Life Meaning, Perceived Discrimination, and Disability Denial. Survey data were analyzed using descriptive statistics, factor analysis, reliability analysis, and hierarchical multiple regression.

### Measurement of disability narrative frames

In Stage 1, we used framing analysis to refine the narrative frames of traditional media and social media platforms and include them as key independent variables in the survey. Framing analysis allows scholars to better understand how media represent content. The definitions of frames by various scholars such as Entman, Gamson and Modigliani, Gitlin, and Iyengar (Matthes, 2009; Zhang and Kraus, 1995) have been frequently cited. Among them, Entman's definition is the one most frequently cited in communication studies (Matthes, 2009; Yang and Chen, 2019). This study on disability also adopts Entman's definition of frames, for example, frames are the selection of some aspects of a perceived reality and their emphasis in a communicating text to “promote a particular problem definition, causal interpretation, moral evaluation, and/or treatment recommendation for the described object (Entman, 1993).

The media corpus included two major national state media organizations representing newspaper and television, the *People's Daily* and *China Central Television* (CCTV), and three social media platforms frequently visited by people with disabilities: Zhihu, Bilibili, and Douyin (Chinese TikTok). Searches of the WiseNews database using Chinese disability-related terms, including “*canji*” (残疾, disability or impairment), “*canzhang*” (残障, disability or barrier), and “*canfei*” (残废, a derogatory term roughly meaning “crippled”), the research team retrieved 1,576 items from *People's Daily* and CCTV. For the dataset of social media platforms, the same terms were also used by the researchers to identify accounts belonging to users with disabilities, after which 1,088 publicly available posts from their profile pages were collected for analysis.

Frame identification combined a deductive-inductive analysis guided by disability models with quantitative content coding. Deductively, established disability models informed a set of preliminary frame categories. Inductively, the research team repeatedly read the media texts and, following Entman's (1993) four framing functions, issue define, causation, moral judgment, and remedies, revised these categories and developed a codebook specifying frame definitions, indicators, and coding rules. Two coders pilot-coded a 5% subsample for each pre-test. Following two rounds of coder training, discussion, and codebook revision, intercoder reliability (Krippendorff's α) reached 0.897, after which the two coders independently coded separate portions of the remaining corpus; multiple frames were allowed from each unit of analysis. The finalized codebook includes six sets of disability frames. Frame frequencies were calculated by media-source type as supplement materials to represent the characteristics of the two media environments (Table 1).

**Table 1:** Frequency of frames and sub-frames refined in both traditional and new media.

Frame	Media type	Issue define	Causation	Moral judgment	Remedy
		Misfortune	Destiny	Hopeless	Suffer
**Traditional**	Traditional media	244	169	138	191
	New media	340	322	355	380
		Vulnerability	No assistance	Inconsistency	Charity
**Charity**	Traditional media	451	463	389	455
	New media	338	382	391	248
		Disease	Impairments	Incapacity	Adjust
**Medical**	Traditional media	389	393	342	402
	New media	461	404	407	350
		Equality	Barriers	Inadequate	Efforts
**Rights**	Traditional media	64	66	39	58
	New media	342	451	354	339
		Difficulty	Hardship	Challenging	Fighting
**Inspiration**	Traditional media	608	555	649	586
	New media	531	459	583	498
		Discrimination	Exclusion	Unacceptable	Understanding
**Relationship**	Traditional media	63	56	60	65
	New media	376	322	419	366

In Stage 2, this study applied these refined frames to develop the survey items and administered a questionnaire survey among communities of people with disabilities (Table 2). For example, the “misfortune” sub-frame identified under the *traditional* frame in Table 1 was operationalized in Table 2 as the item “Disability is a misfortune for me and my family” to assess respondents' endorsement of the traditional narrative. The respondents were asked to confirm their recognition of each option from “strongly disagree,” “disagree,” “half and half,” “agree,” to “strongly agree “according to Likert's 5-point scale. After the factor analysis and reliability test, all six sets of the frame passed the reliability and validity test. Six frame factors were generated (now referred to as frame packages), with factor loadings ranging from 0.587 to 0.882. Cronbach's alpha coefficients ranged from 0.722 to 0.863 (Table 2). We separately labeled these factors as *traditional* frame package, *charity* frame package, *medical* frame package, *inspirational* frame package, *relationship* frame package, and *rights* frame package.

**Table 2:** Factors of narrative frames and the results of reliability and validity tests.

	Items developed by frames	Factor loading	Mean
Traditional frame package			
Issue define	Disability is the misfortune of myself and my family.	0.826	2.99
Causation	Disability is because of my miserable destiny.	0.882	2.43
Moral judgment	My biggest fear is that I am trapped in the current situation.	0.818	2.60
Remedy	Disability cannot be changed; I have to accept it, and live in suffering.	0.846	2.11
	KMO = 0.783; Cronbach's alpha=0.863		
Medical frame package			
Issue define	Disability is a kind of physical or psychological disease.	0.830	3.13
Causation	Disability is caused by the impairments.	0.810	3.40
Moral judgment	My biggest trouble is that I do not have a functional body.	0.658	2.81
Remedy	If provided with medical advice or adjustments, I can be cured.	0.650	3.17
	KMO = 0.623; Cronbach's alpha = 0.722		
Relationship frame package			
Issue define	Disability is a type of discrimination by mainstream society.	0.790	3.34
Causation	Disability is caused by the social exclusion of people by the mainstream society.	0.870	3.24
Moral judgment	My biggest trouble is that my ability and values have been denied.	0.861	3.12
Remedy	If there are more people encouraging and understanding me, I can have a better life.	0.798	3.49
	KMO = 0.773; Cronbach's alpha = 0.850		
Charity frame package			
Issue define	People with disabilities are vulnerable and need help.	0.661	2.83
Causation	Disability is caused by the undeveloped welfare.	0.815	3.27
Moral judgment	My biggest concern is that I cannot receive constant assistance from others.	0.804	3.16
Remedy	If the government, society, and charity institutions can provide more economic assistance and care, I can be better.	0.820	3.28
	KMO = 0.774; Cronbach's alpha = 0.779		
Inspirational frame package			
Issue define	Disability is a difficulty that I need to overcome	0.723	3.66
Causation	Like everyone else; disability is a harsh time in one's life.	0.794	3.41
Moral judgment	My biggest trouble is that I cannot get better immediately	0.587	2.87
Remedy	I believe that if I constantly fight against disability, my life can be better.	0.644	4.11
	KMO = 0.774; Cronbach's alpha = 0.730		
Rights frame package			
Issue define	People with disabilities should have the same human rights as those without disabilities.	0.610	4.63
Causation	Disabilities are caused by the lack of personalized barrier-free facilities.	0.829	4.14
Moral judgment	The biggest trouble is that my basic needs and demands cannot be satisfied.	0.760	3.91
Remedy	Through the efforts of government and the society, I can have a better life.	0.833	3.88
	KMO = 0.744; Cronbach's alpha = 0.758		

The *traditional* package can be explained in terms of the moral model of disability; the *charity* and *medical* packages are both tragedy-based narratives that fit the *charity* and *medical* models of disability research, respectively. The *relationship* and *rights* packages follow the logic of the social model, with the *rights* factor paying more attention to disability equality, such as a barrier-free environment and other facilities, and the *relationship* packages emphasizing attitudinal perspectives such as anti-discrimination, prejudice, and stereotyping. The *inspirational* package views people with disabilities as “heroes” who overcome their impairment. However, this *inspirational* narrative does not conform to the affirmation model described above. Despite the positive tone, the *inspirational* framework still emphasizes how people with disabilities “fight” impairments rather than being proud of and accepting of their disability.

### Measurement of disability identity

Identity can help people understand different parts of the self. Few systematic quantitative studies have examined disability identity Based on the research of previous scholars such as Putnam (2005), Dunn and Burcaw (2013), disability researcher Zapata (2019) has attempted to examine a number of measurement indicators of identity for people with disabilities through empirical investigation. In Zapata's study, the scholar applied both positive and negative indicators within his measurement scales and summarized a set of indicators that can generate four factors of disability identity, which were named Self-Worth, Positive Personal Meaning, Pride/Affirmation, and Acceptance.

Zapata's measurement model provides a theoretical basis for our research. We used the items developed by Zapata in the United States as a baseline upon which we developed the questionnaire for our study. Because the original measure was developed in English, its items were translated and adapted for use with Chinese respondents. The research team and two disability-community leaders reviewed the translated items for semantic clarity and cultural relevance. Zapata's candidate items were translated and culturally adapted to ensure semantic clarity and relevance to Chinese disability communities; no entirely new identity items were introduced. For example, the item “There is nothing worse than having a disability.” was adapted in Chinese to convey “Having a disability is the worst thing,” because a literal translation of “nothing worse than” would sound unnatural in Chinese grammar; this revision improved linguistic clarity without altering the underlying construct.

Participants were asked to choose the response that best described their situation, with each item rated on a 5-point Likert scale ranging from “mostly disagree” (1) to “mostly agree” (5). Exploratory factor analysis and reliability analysis were conducted to examine the dimensional structure and internal consistency of the adapted measure. Items with factor loadings of at least 0.50 were retained. The final solution retained 21 items with loadings ranging from 0.528 to 0.848 and yielded four dimensions, although their structure differed from Zapata's original model. Four positively worded items formed the Self-worth dimension (Cronbach's α= 0.825), whereas four negatively worded self-worth items formed a separate Perceived Discrimination dimension (α= 0.813). Items originally measuring Pride/Affirmation loaded together with the Positive Personal Meaning items, producing an eight-item Life Meaning dimension (α= 0.900). The remaining five worded items formed the Disability Denial dimension (α= 0.871). Scores for these four dimensions were subsequently entered as dependent variables in the regression analyses (Table 3).

**Table 3:** Self-worth, life meaning, discrimination, and self-denial in disability identity.

Aspects of identity	Factor loading	Mean
Self-worth		
People with disabilities can be productive contributors of society.	0.811	4.42
As a person with disability, I have plenty to offer my family or friends.	0.811	4.09
I deserve to be valued as much as people without disabilities.	0.829	4.06
I have as much to offer the world as people without a disability.	0.799	4.13
KMO = 0.824; Cronbach's alpha = 0.825		
Perceived Discrimination		
Due to my disability, I feel that I do not have anything to offer to others.	0.848	1.83
Due to my disability, I feel worthless.	0.835	1.62
Due to disability, I regard myself as others' burden.	0.846	2.40
Due to my disability, I feel inadequate in my relationships.	0.698	3.14
KMO = 0.824; Cronbach's alpha = 0.813		
Life meaning		
I have discovered benefits to having a disability.	0.802	3.62
I have grown as a person because of my disability.	0.798	3.71
My disability has made me a stronger person.	0.782	3.77
Considering the challenges that I have faced as a person with a disability, I have a lot of respect for myself.	0.728	3.55
There are good things about having a disability.	0.715	3.93
My disability has given me an appreciation for life.	0.714	3.53
As a person with a disability, I possess skills and strengths that people without disabilities do not.	0.637	3.47
My disability gives me perspective on what matters in life.	0.528	3.80
KMO = 0.901; Cronbach's alpha = 0.900		
Disability Denial		
Due to my disability, I will never become the person I want to be.	0.685	2.76
Due to my disability, I will never achieve my goals in life.	0.667	2.40
Due to my disability, I do not pursue my dreams.	0.627	2.91
There is nothing worse than having a disability.	0.617	2.49
My disability prevents me from living a meaningful life.	0.583	2.61
KMO = 0.901; Cronbach's alpha = 0.871		

### Measurement of sociodemographic and media use

Other measuring variables included media use and sociodemographic indicators. We used six variables to measure sociodemographic background: the impairment severity (from “not at all severe” to “very severe”), time of onset of disability (initial or not), family income (from “very low” to “very high”), personal income (from “less than 0” to “very high”), level of socioeconomic status in the local community (from “very high” to “very low”), and educational level.

Media use includes the channels through which one can access disability information (television, newspaper, social media), the concern of media's judgment on disability (from “rarely” to “very high”), participation in the discussion of social media (from “rarely” to “always”), and willingness to reveal disability identity in social media (from “rarely” to “always”).

### Survey sampling

Given the specificity of the sample we wanted to study, we relied on a nonprobability sample by inviting survey participants from online disability communities. We identified two online community organizers who were part of institutions: the *Disabled Persons' Federation Association* and a training foundation for people with disabilities. These two individuals had previously received training on disability empowerment. After we explained the purpose and design of the study, the two online community leaders were asked to join the research team. They then helped refine the questionnaire and select qualified interviewees from their groups and other large disability communities. Candidates were not selected based on their level of disability, category, age, or gender.

The selected participants were asked to answer all questions based on their own experience. A member of the research team verified that the questionnaire had been properly completed before rewarding the participant. After excluding several questionnaires that did not meet the requirements of the study for various reasons (e.g., users without disabilities, too many missing answers, and the same answer throughout the questionnaire), we included 332 valid questionnaires in the analysis. All study participants gave informed consent prior to their participation.

## Results

### Descriptive results

A total of 332 valid questionnaires were collected, including 162 male (48.8%) and 167 female (50.3%), three identified with another gender category (0.9%). Of the participants, 40.1% (133) were people with physical impairments, 47.3% (157) were people with visual impairments, followed by 12 (3.6%) with hearing impairments, 12 (3.6%) with intellectual impairments, and 18 (5.4%) with other disability categories. Regarding the degree of impairment, 220 (66.3%) of the participants had impairments that were apparent at first glance; 210 (63.3%) classified their impairments as severe. 183 (55.1%) of the participants required their disability initially.

Regarding family income, 73.8% (245) reported a monthly family income of less than 10,000 RMB (approximately 1,428 USD); and 54.5% (181) of participants reported a monthly family income of less than 5,000 RMB (approximately 714 USD). In terms of personal income, 82.8% (275) of respondents reported that their monthly income was less than 5,000 RMB. 19.3% (64) had monthly family income less than 0 RMB (unemployed and need to pay medical expenses); 30.7% (102) reported low economic status in their community, 34.0% (113) reported lowest economic status in their community. Participants also reported their education level: 29.8% (99) had high school or lower degrees, 50.9% (169) had college degrees.

Finally, regarding media use, 21.7% (72) of respondents indicated that they frequently or very frequently use television for information about disabilities, 21.1% (70) frequently read the newspaper as a source of information (including the online version), 83.7% (278) frequently visit social media for information. Referring to social media engagement, the results indicated that 50.9% (169) of respondents usually participate in discussions related to disability topics; 54.5% (181) are willing to reveal their disability identity on social media, and 70.5% (234) pay significant attention to the judgments on disabilities.

### Disability narratives and identity

Hierarchical multiple regression models were estimated separately for the four dimensions of disability identity: Self-worth, Life Meaning, Perceived Discrimination, and Disability Denial. Predictors were entered in three blocks. The first block comprised six sociodemographic variables, the second comprised four media-use variables, and the third comprised four disability narrative-frame packages: *traditional, charity, inspirational*, and *rights*. Although six frame packages were examined in the survey, the *medical* and *relationship* packages were not statistically significant in the regression model, therefore were removed from the analysis. Table 4 presents the results of the four models.

**Table 4:** Hierarchical regression results for the associations of sociodemographic characteristics, media use, and narrative frames with disability identity.

Sociodemographic background	Self-worth	Perceived Discrimination	Life meaning	Disability Denial
Impairment severity	0.124^*^	−0.040	0.089^*^	0.090^*^
Disability on set	−0.006	−0.055	0.117^**^	0.080^*^
Education level	0.035	−0.066	−0.007	−0.128^**^
Family income	−0.069	−0.050	−0.074	−0.133^*^
Personal income	0.110^*^	−0.047	0.009	0.139^*^
Local socioeconomic status	−0.133^*^	0.000	−0.132^**^	−0.018
*R square change*	*0.090*	*0.095*	*0.069*	*0.124*
Media use				
Television news	−0.046	−0.004	0.126^**^	0.101^*^
Concern on judgment about disability	0.042	−0.006	0.042	−0.095^*^
Participate in online discussion	0.126^**^	−0.109^*^	−0.006	−0.076^*^
Willingness to reveal disability status	0.076	−0.174^***^	0.153^**^	0.065
*R square change*	*0.061*	*0.061*	*0.092*	*0.041*
Narrative frames				
Traditional frame	−0.300^***^	0.405^***^	−0.325^***^	0.351^***^
Charity frame	−0.074	0.150^*^	−0.116^*^	0.179^**^
Inspirational frame	0.205^***^	−0.112^*^	0.377^***^	0.116^*^
Rights frame	0.265^***^	0.099	0.095^*^	0.094
*R square change*	*0.187*	*0.261*	*0.233*	*0.249*
*R square*	*0.337*	*0.417*	*0.394*	*0.414*

^***^p < 0.001. ^**^p < 0.01. ^*^p < 0.05.

As shown in Table 4, the regression model for Self-worth was statistically significant (*p* < 0.001, R^2^ = 0.34), as was the model for Life Meaning (*p* < 0.001, R^2^ = 0.39). Higher scores of the *inspirational* and *rights* frame packages were associated with higher Self-worth (*inspirational*: β = 0.205, *p* < 0.001; *rights*: β = 0.265, *p* < 0.001) and Life Meaning (*inspirational*: β = 0.377, *p* < 0.001; *rights*: β = 0.095, *p* < 0.05). Conversely, higher scores of the *traditional* frame package were associated with lower Self-worth (β = −0.300, *p* < 0.001) and Life Meaning (β = −0.325, *p* < 0.001). The *charity* frame package was also negatively associated with Life Meaning (β = −0.116, *p* < 0.05), although its association with Self-worth was not statistically significant. Overall, the tragedy-oriented frames tended to be associated with less positive dimensions of disability identity, whereas the *rights* frame and the positively toned *inspirational* frame showed positive associations with these dimensions.

The regression model for Perceived Discrimination was also statistically significant (*p* < 0.001, R^2^ = 0.42). Higher scores of the *traditional* (β = 0.405, *p* < 0.001) and *charity* (β = 0.150, *p* < 0.05) frame packages were associated with higher Perceived Discrimination, whereas higher scores of the *inspirational* frame package was associated with lower Perceived Discrimination (β =-0.112, *p* < 0.05). The model for Disability Denial was likewise statistically significant (*p* < 0.001, R^2^ = 0.41). The *traditional* (β = 0.351, *p* < 0.001), *charity* (β = 0.179, *p* < 0.01), and *inspirational* (β = 0.116, *p* < 0.05) frame packages were positively associated with this dimension. The *rights* frame package was not significantly associated with either Perceived Discrimination or Disability Denial.

Comparing the patterns across the four dimensions of disability identity, greater endorsement of the tragedy-oriented frames, particularly the *traditional* and *charity* frames, was associated with higher Perceived Discrimination and lower acceptance, as well as lower Self-worth and Life Meaning. By contrast, the *rights* frame was positively associated with Self-worth and Life Meaning but was not significantly associated with Perceived Discrimination or Disability Denial. The *inspirational* frame package showed a more ambivalent pattern. It was positively associated with Self-worth and Life Meaning and negatively associated with Perceived Discrimination, but it was also positively associated with Disability Denial, indicating a paradox in disability acceptance. This mixed pattern may reflect the tragedy-oriented element that remains embedded in *inspirational* frame, which emphasize “heroes” overcoming impairment and adversity. Thus, participants who endorsed *inspirational* narratives may report more positive evaluations of their value and life meaning while simultaneously experiencing greater difficulty accepting disability as part of their identity.

### Traditional media, digital platforms and identity

With regard to the associations of media use and social background with disability identity, the regression analyses identified significant relationships between several sociodemographic and media-use variables and the four dimensions of disability identity.

Table 4 shows that greater impairment severity (β = 0.124, *p* < 0.05), higher personal income (β = 0.110, *p* < 0.05), higher perceived local socioeconomic status within the local community (β = −0.133, *p* < 0.05), and more frequent participation in online disability-related discussions (β = 0.126, *p* < 0.01) were associated with higher Self-worth. Greater impairment severity (β = 0.089, *p* < 0.05), acquired rather than congenital disability (β = 0.117, *p* < 0.01), higher perceived local socioeconomic status (β = −0.132, *p* < 0.01), more frequent use of television as a source of disability-related information (β = 0.126, *p* < 0.01), and greater willingness to disclose disability identity on social media (β = 0.153, *p* < 0.01) were associated with higher Life Meaning. Because local socioeconomic status was coded from high (1) to low (5), its negative coefficients indicate that higher socioeconomic status was associated with higher Self-worth and Life Meaning.

Regarding Perceived Discrimination, more frequent participation in online disability-related discussions (β = −0.109, *p* < 0.05) and greater willingness to disclose disability identity on social media (β = −0.174, *p* < 0.001) were associated with lower Perceived Discrimination. Regarding disability denial, greater impairment severity (β = 0.090, *p* < 0.05), acquired disability (β = 0.080, *p* < 0.05), higher personal income (β = 0.139, *p* < 0.05), and more frequent television use (β = 0.101, *p* < 0.05) were associated with higher scores on this dimension. By contrast, higher educational attainment (β = −0.128, *p* < 0.01), higher household income (β = −0.133, *p* < 0.05), greater attention to media evaluations of disability (β = −0.095, *p* < 0.05), and more frequent participation in online disability-related discussions (β = −0.076, *p* < 0.05) were associated with lower scores.

Taken together, the results reveal differentiated associations across the four dimensions of disability identity. Greater impairment severity and acquired disability were associated with higher Life Meaning but also with greater Disability Denial, indicating that their associations with disability identity were not uniformly positive or negative. Television use showed a similarly mixed pattern, as it was associated with both higher Life Meaning and greater Disability Denial. In contrast, participation in online disability-related discussions was associated with higher Self-worth, lower Perceived Discrimination, and greater acceptance, while willingness to disclose disability identity on social media was associated with higher Life Meaning and lower Perceived Discrimination. These patterns suggest that traditional media use, digital-media participation, and sociodemographic characteristics correspond differently with the multiple dimensions of disability identity.

## Discussion and conclusions

Disability identity is a multidimensional construct situated at the intersection of individual experience, impairments, and available narrative resources (Johnstone, 2004). The present study demonstrates that disability narratives circulating through traditional media and social media platforms are differentially associated with Self-worth, Life Meaning, Perceived Discrimination, and Disability Denial. These relationships also vary with impairment severity, educational attainment, income, and perceived socioeconomic status, highlighting the heterogeneity of disability identity.

People with disabilities who accept the negative messages might develop a stigmatized aspect of disability in their lives, while those who develop a closer connection to their online media sources may consciously counteract the stigma and develop a positive disability identity with elements of positive self-worth and a positive sense of life. The key element is that tragic narratives are “invisible barriers” and labeling causes people with disabilities to feel “shame” when they internalize or “buy into” these discourses. Eventually, people with disabilities may face exclusion or prejudice, which can lead to negative feelings of self, such as Perceived Discrimination and Disability Denial.

The *rights* frame package is a kind of legitimizing narrative that serves politicized identity as a communicative tool that asserts that a marginalized group is equal in the larger society, whereby an individual's personal responsibility for his or her identity cannot be understood merely as a reaction to trauma or tragedy, but must be located within a framework that is both socially and politically accounted for. Such demands are intended to provide a countervailing force to delimiting views of people with disabilities and oppressive policies by focusing on the humanities and on personal and political issues or individuals' adoption of ideology rather than “curing” the problem of disability. To put it better: The identity of people with disabilities is gradually being formalized in social structures around the world.

This study also notes a paradox of disability identity, e.g., the narrative of *inspirational* rejects discrimination but does not lead to acceptance of identity. Such a paradox may result from the narrative of “heroism” that overcomes or challenges imperfection, failure to control the body, vulnerability to weakness, pain, and death. It is an identity assigned by others or portrayed by the media in a positive tone, rather than a narrative based on the affirmation model of disability. Affirming a disability is a way of feeling included in society, having the same rights and responsibilities as other citizens, being recognized and treated like everyone else in a group or society at large. This can be explained by one of our disability community participants who told us the following when we created the survey, “I read a lot of news and also actively participate in online discussions. I know that many of the words you have told us are right, but it is difficult for me to do that.” Additionally, he asked: “How can I feel my life worth living when I am in such a desperate condition? I am uncomfortable when you talk about disability pride. I feel like you are laughing at me, or you are discriminating against me!”

This expression foreground the conceptual distinction between positive valence and affirmative identity. Rejecting stigmatizing classifications or adopting positively coded subject positions does not necessarily displace the impairment-centered assumptions through which disability is devalued. Within the Chinese media environment, the discursive repertoire available for disability identification remains asymmetrical. Deficit-oriented and inspirational frames are readily recognizable, whereas culturally embedded narratives that recognize disability as a legitimate and valued form of human difference remain comparatively limited. This asymmetry constrains the narrative resources through which people with disabilities can reconcile disability pride with their material circumstances and incorporate disability into a coherent self-concept. Affirmative identity formation consequently requires narrative resources that legitimize disability without invoking cure, compensation, or exceptional achievement.

In addition to disability narratives and media use, the identity of people with disabilities is also influenced by other factors: educational attainment, family, and socioeconomic status. The narratives show that people with disabilities often cross socioeconomic class lines and thus play a role in constructing hegemonic policies and stereotypes toward people with disabilities. In general, people from higher socioeconomic classes had more opportunities to view themselves positively, namely, higher socioeconomic factors lead to positive self-worth and a positive sense of purpose in life; higher family income and education lead to higher acceptance. One exception is that higher personal income decreases acceptance. This could be because the person whose disability is required needs more support to be accepted.

This study still has some limitations. First, some specific communities of people with disabilities reject the term “disability” and disability identity. Deaf activists, for example, claim that people who do not hear and use sign language are a linguistic minority and not a population with disabilities; therefore, they declined to participate in the project. In addition, we did not discuss the impact of different disability platforms on the identity development of people with disabilities in a more nuanced information context. Finally, we did not consider people with disabilities who do not use social media. Although our generalizations are overly simplistic, further studies reflecting the heterogeneity of disability identity are needed to understand the predictors of disability identity. Nevertheless, the idea that different worldviews are important to society is significant. We hope that the design of the narrative framework and media use in this study could help society name and resolve common issues and barriers that block a positive self-concept of disability.

## Data Availability

The raw data supporting the conclusions of this article will be made available by the authors, without undue reservation.
